# Oxford Textbook of Oncology, Second Edition

**DOI:** 10.1038/sj.bjc.6600425

**Published:** 2002-08-12

**Authors:** J O Armitage

**Affiliations:** University of Bebraska Medical Center, Omaha, USA

## Abstract

doi:10.1038/sj.bjc.6600425 www.bjcancer.com

© (2002) Cancer Research UK

## 

The second edition of the ‘Oxford Textbook of Oncology’, edited by Souhami, Tannock, Hohenberger and Horiot, is a well presented, thorough review of the field of oncology. The book is interdisciplinary with important contributions from medical, surgical, and radiation oncologists. Although the two volumes make it somewhat less convenient to use, the material is not difficult to find, and the chapters are generally well written.

This is a largely European text. It is more comprehensive than the ‘Textbook of Medical Oncology’ by Cavalli, Hansen, and Kay, although that text is well-written and easy to use. The ‘Oxford Textbook of Oncology’ deals with subjects in more depth and is more likely to yield an unusual fact about a particular cancer.

The 2nd edition of the ‘Oxford Textbook of Oncology’ is in many ways like two major American texts – ‘Cancer, Principles and Practices of Oncology’ by DeVita, Hellman and Rosenberg, and ‘Clinical Oncology’ by Abelhoff, Armitage, Lichter, and Niederhuber. Table 1

**Table 1:**
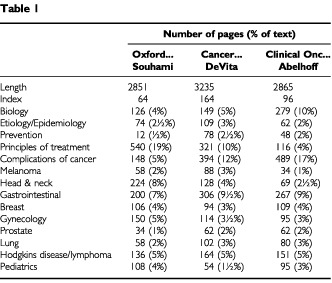

compares and contrasts several aspects of these texts.

These three texts are all similar in length. The DeVita textbook is the most extensively indexed and the Souhami text the least extensively indexed. The Abelhoff text has more emphasis on biology than either DeVita or Souhami. The Souhami text deals less with prevention but much more with the principles of the various therapeutic modalities. However, it deals less with the clinical problems associated with cancer and metastasis.

The varying emphasis on the different malignancies among these texts reflected in the length of the disease specific chapters is interesting. It is uncertain whether this represents a different emphasis of the editors, or simply a proclivity to long chapters by certain of the authors. Either way, head and neck cancer is much more extensively dealt with in the Souhami text than in the other two, and pediatrics less in the DeVita text. In many areas (e.g. lymphoma, lung cancer, and breast cancer), similar proportions of the books are devoted to the topic.

One problem with any oncology text is the difficulty in incorporating new advances. For example, imatinib mesylate has become the treatment of choice for chronic myeloid leukemia and unresectable gastrointestinal stromal sarcomas. However, the drug is dealt with very briefly in the chronic myeloid leukemia chapter and not at all in the sections on gastrointestinal stromal sarcomas. Also, one of the most important new techniques in classifying, defining prognostic groups and, eventually, developing new treatments is likely to be the use of gene arrays. This topic is addressed briefly in the chapter on genetics and not at all in the chapters on the diseases where it is likely to be soon applied in the clinic. This is to some degree an unavoidable problem and one that will certainly be dealt with in future editions.

In summary, Souhami *et al* have produced a high quality textbook of oncology. By owning all three of the major texts, there would be very few facts one could not find. However, these are all excellent resources.

